# Establishing the relationship of inhaler satisfaction, treatment adherence, and patient outcomes: a prospective, real-world, cross-sectional survey of US adult asthma patients and physicians

**DOI:** 10.1186/s40413-015-0075-y

**Published:** 2015-09-10

**Authors:** David Price, Brooke Harrow, Mark Small, James Pike, Victoria Higgins

**Affiliations:** Professor of Primary Care Respiratory Medicine, Academic Primary Care, Division of Applied Health Sciences, University of Aberdeen, Polwarth Building, Aberdeen, AB25 2ZD UK; Meda Pharmaceuticals, 265 Davidson Avenue, Suite 400, Somerset, NJ 08873-4120 USA; Adelphi Real World, Adelphi Mill, Bollington, Macclesfield, Cheshire, SK10 5JB UK

**Keywords:** Adherence, Allergic rhinitis, Asthma, Inhaler device, Patient outcomes, Treatment satisfaction

## Abstract

**Background:**

Inhaled asthma medications are the mainstay of treatment for chronic asthma. However, nonadherence rates for long-term inhaler therapy among adults are estimated to exceed 50 %. Nonadherence is associated with unfavorable clinical outcomes and diminished quality of life. Research suggests that adherence is associated with patients’ satisfaction with their treatment regimen and other factors, such as concomitant allergic rhinitis and tobacco use.

**Methods:**

This prospective, cross-sectional survey of physicians and their patients evaluated the relationship between patient satisfaction with attributes of inhaler devices, treatment adherence, and clinical outcomes. Primary care and specialist physicians completed a physician-reported patient record form for patients with a confirmed asthma diagnosis. Patients for whom a physician-reported form was completed were invited to complete a patient-reported form. Both surveys collected information about demographics, symptoms, exacerbation history, treatment, smoking status, comorbidities, type of inhaler device, and treatment adherence. Patients also indicated the degree to which they were satisfied with attributes of their currently prescribed inhaler device(s). Partial least squares path modeling quantified relationships between latent variables and clinical outcomes.

**Results:**

A total of 243 patients were included in our analysis and 41 % had poorly controlled asthma. More favorable clinical outcomes were significantly associated with greater patient satisfaction with drug delivery (*P* = 0.002), higher medication adherence (*P* = 0.049), no history of tobacco use (*P* < 0.001), and absence of comorbid allergic rhinitis (*P* = 0.005). Attributes associated with device satisfaction included patient perceptions of consistency in the amount of drug delivery to the lungs, ease of use, and feedback about the number of remaining doses.

**Conclusions:**

Higher patient satisfaction with their asthma drug delivery inhaler device is a significant predictor of more favorable clinical outcomes while allergic rhinitis and smoking history were negatively associated with optimal control of asthma. These findings provide clinicians with opportunities to improve patients’ clinical outcomes by tailoring choice of inhaler device therapy and providing education about the correct way to use the device to ensure optimal outcomes. Patients will likely benefit from medical therapy to manage comorbid allergic rhinitis and smoking cessation interventions. Patients unable to stop smoking may require alternative medical therapies to improve their clinical outcomes.

## Introduction

Inhaler therapy is the cornerstone of treatment for asthma, with pressurized metered dose inhalers and dry powder inhalers the most frequently used inhalation devices [1, 2], with numerous drug-inhaler combinations currently available to prescribers and patients [3]. Despite the efficacy of inhaled asthma medications, it is estimated that 50 % of adults and children on long-term therapy for asthma fail to adhere to their treatment regimen [4], with one study reporting that 84.6 % of patients demonstrated some degree of nonadherence [5]. Among older adults with asthma, 57 % demonstrated poor medication adherence [6].

Nonadherence is considered a key predictor for the failure of patients to attain and maintain their treatment goals, which results in poor health and quality of life outcomes in asthma [7] as well as other chronic diseases [8]. While adherence plays a critical role in achieving optimal outcomes for patients with asthma, other factors that may affect symptom control also merit consideration. Specifically, poor asthma control is associated with concomitant allergic rhinitis (AR) [9–14], and less favorable outcomes are reported for patients who have concomitant AR [15–18]. Current use of tobacco also compromises the outcomes of patients with asthma, [10, 19–23] with evidence to suggest that active smoking is associated with symptom exacerbation, more rapid declines in lung function [20, 24, 25], and a reduced response to inhaled corticosteroids (ICS) [25–28]. These findings have led to the recognition that patients with asthma who also use tobacco products may require treatment regimens that target inflammation of the small airways [21].

Research suggests that treatment adherence among patients with chronic health conditions, including asthma, is predicted, in part, by patients’ satisfaction with their treatment regimen [6, 29–32]. Several studies report a positive relationship between adherence to asthma medications and treatment satisfaction [6, 31, 33], as well as in other chronic diseases [32]. Satisfaction with asthma management was 1 of 7 themes identified in a factor analysis of items that were significantly associated with adherence [33]. A cross-sectional study assessed physicians’ perceptions of patient adherence with administration frequency and inhaler device usage and patient satisfaction ratings for 13 attributes of their inhaler device. Notably, higher adherence ratings were associated with greater patient-reported satisfaction with their device [31].

This research was undertaken to identify the relationship among satisfaction with attributes of inhaler devices, treatment adherence, and clinical outcomes in adult patients with asthma. In particular, we sought to expand on earlier research [31] by examining relationships among patient satisfaction with specific attributes of inhaler devices and treatment adherence on measures of asthma control and overall health status. We also evaluated the impact of comorbid AR and tobacco use on the same clinical outcomes in this patient population, 2 factors also deemed to be modifiable through appropriate treatment adjustment or change in exposure.

## Methods

### Study design

This was a prospective, cross-sectional survey of physicians and their patients, with data collected through the US 2013 Adelphi Disease Specific Program (DSP). A complete description of the methods of the DSP has been previously published [34]. Eligible physicians completed a physician-reported patient record form for the next 5 consecutive patients with a physician-confirmed diagnosis of asthma regardless of the reason for the visit. The physician-reported forms reflected physicians’ knowledge about the health status of patients seeking routine care. Patients for whom a physician-reported form was completed were invited to complete a patient-reported form, with no suggestions from nurses or the physician.

All diagnostic test and treatment decisions were made at the discretion of each physician with no tests, treatments, or investigations performed as part of this prospective, observational, cross-sectional survey. The survey was performed in full accordance with the US Health Insurance Portability and Accountability Act (HIPAA) 1996 [35]. Each patient consented to anonymous, aggregated reporting of research findings as required by the HIPAA guidelines. The physician-reported form and the patient-reported form were de-identified with unique identification numbers assigned to physicians and patients that allowed linkage of a patient-reported form with the corresponding physician-reported form.

### Subjects

Physicians were identified from lists that were publically available and contacted by telephone to determine their eligibility for study participation. Physicians were eligible to participate in this research if they had completed their medical training between 1978 and 2008, were personally responsible for treatment decisions and management of patients with asthma, and saw a minimum of 3 patients with asthma per week. Survey data were also collected from adult patients with asthma who consulted with primary care physicians, pulmonologists, or allergists between September and December 2013. Eligibility criteria for patients included age ≥12 years and a physician-confirmed diagnosis of asthma with no comorbid chronic obstructive pulmonary disease. Patients were included in the analysis if they were on one inhaled maintenance therapy and completed all of the questions on the patient-reported form regarding inhaler device satisfaction, adherence, asthma control, sleep quality, and quality of life.

### Assessments

The physician-reported form obtained information on approximately 130 patient and disease variables including: demographics; symptoms; exacerbation history; treatment; smoking status; comorbid health conditions; physician visits; type of prescribed inhaler device; lung function test results; and perceptions of patients’ disease knowledge, engagement, and adherence. Completion of the physician-reported form required approximately 15 min per patient and physicians were compensated for their participation.

The patient-reported form included items similar to those on the physician-reported form as well as assessment of satisfaction with their currently prescribed inhaler device(s), which could include multiple devices for maintenance therapy. Patients were not asked to indicate their satisfaction relating to specific device(s) they were prescribed (eg, pressurized metered dose inhaler, dry powder inhaler) based on evidence that patients are unable to accurately report the names and dosages of medications, particularly when they take many medications, are cognitively impaired, or have low health literacy [36, 37]. Rather, they were asked to indicate their overall satisfaction with 12 attributes of all their current inhaler device(s), such as consistency of drug delivery, simplicity of instructions for use, and information about number of remaining doses. While the questionnaire has not undergone formal psychometric evaluation, it has been used for the last 7 years by the Adelphi DSP being administered to 5006 patients. In addition, expert consultants reviewed and guided the selection and wording of questions based on their experience, knowledge of the disease state, and the published literature. The instrument has also been used in previous research to evaluate relationships between inhaler device satisfaction, patient adherence, and health outcomes [31]. The 12 attributes were grouped into 3 domains relating to drug delivery, device functionality, and device feedback. The decision to group the 12 attributes into these 3 domains was guided by expert consensus and disease knowledge. Satisfaction ratings were assessed on a 5-point Likert scale with a score of 1 indicating not at all satisfied and a score of 5 indicating very satisfied for each device feature.

Adherence to current maintenance treatment was measured with the 8-item Morisky Medication Adherence Scale (MMAS-8) [38]. Patients also completed the Asthma Control Test (ACT), Jenkins Sleep Evaluation Questionnaire (JSEQ), and quality of life assessment on the EuroQol-5D-3L and (EQ-5D-3L). The ACT is a 5-item questionnaire that assesses recall of symptoms and daily functioning during the past 4 weeks, with scores exceeding 19 indicative of well-controlled asthma [39]. The JSEQ is a validated 4-item survey assessing quality of sleep in the preceding 4 months, with scores ranging from 0 to 20 and higher scores associated with worse sleep quality [40]. The EQ-5D-3L is also a validated, standardized instrument that assesses 5 life dimensions, including mobility, self-care, usual activities, pain/discomfort, and anxiety/depression. Results indicate 1 of 3 levels of severity ranging from no problems to some or moderate problems, or extreme problems within each of the 5 dimensions [41]. On average, it took approximately 20 min for patients to complete the patient-reported form and the validated patient-reported outcome (PRO) instruments. Patients received no compensation for completion of the patient-reported form and the PROs.

### Statistical analysis

We calculated descriptive statistics for all variables including percent responses for categorical variables and means with standard deviations for continuous variables. Partial least squares path modeling was used to quantify inner model relationships between latent variables [42]. All statistical analyses were performed with Stata/SE version 13.1 [43] and R version 3.0 [44].

Partial least squares path modeling is a multivariate analysis method that examines a system of linear relationships between multiple sets of variables. It is based on the assumption that each set of variables is represented by a latent variable or theoretical concept. Individual variables within each set of variables are referred to as manifest variables because they are a manifestation of the latent variable or theoretical concept. Seven latent variables were included in the model (Table 1): 1) patient-reported satisfaction with inhaler device drug delivery attributes, 2) patient-reported satisfaction with inhaler device functionality attributes, 3) patient-reported satisfaction with inhaler device feedback attributes (Table 2), 4) comorbid AR, 5) adherence based on the MMAS-8, 6) physician-reported smoking history (ever smoked, total years smoked, and amount smoked per day), and 7) clinical outcomes (physician-reported number of asthma exacerbations in preceding 12 months and PROs on the ACT, JSEQ, and EQ-5D-3L). Exacerbations were defined as a worsening of symptoms that exceeded normal day-to-day variations.

**Table 1:** Latent and manifest variables included in the partial least squares model

Latent Variable	Manifest Variable
Drug delivery satisfaction	Satisfaction: I get the same amount of medicine delivered to my lungs each time.
	Satisfaction: I do not need to breathe in hard to inhale my medicine.
	Satisfaction: Low/no irritation in mouth and throat.
	Satisfaction: I do not need to breathe in at the same time as I press my inhaler.
Device functionality satisfaction	Satisfaction: The instructions are simple and easy to use.
	Satisfaction: It is built to last and will not break easily.
	Satisfaction: No need for me to put the medicine in the inhaler before I use it.
	Satisfaction: Easy to hold and carry around with me.
	Satisfaction: Can reuse the inhaler for more than one month.
Device feedback satisfaction	Satisfaction: It tells me how many doses of medicine I have left.
	Satisfaction: The inhaler locks when empty so it cannot be used anymore.
	Satisfaction: It tells me when my dose of medicine has been inhaled correctly.
Comorbid allergic rhinitis	Physician-reported concomitant allergic rhinitis
Treatment adherence	MMAS-8
Smoking history	Whether patient ever smoked (clinician-reported)
	Clinician-reported cigarettes smoked per day
	Clinician-reported years smoked cigarettes for
Clinical outcomes	Clinician-reported number of asthma exacerbations in preceding 12 months
	ACT score
	JSEQ score
	EQ-5D-3L score

ACT, Asthma Control Test; EQ-5D-3L, EuroQol-5D-3L; JSEQ, Jenkins Sleep Evaluation Questionnaire; MMAS-8; Morisky Medication Adherence Scale

**Table 2:** Demographic and clinical characteristics of patients (*N* = 243)

Characteristic	
Age, years	
• Mean (SD)	40.69 (15.52)
• Range	12.0-78.0
• Median	41.0
• IQR	27.0, 52.0
Gender, *n* (%)	
• Female	132 (54.32)
Ethnicity	
• Caucasian	177 (72.84)
Body mass index, mg/kg^2^	
• Mean (SD)	28.15 (6.85)
• Range	15.21-54.71
• Median	26.58
• IQR	23.34, 31.19
Body mass index, mg/kg^2^, *n* (%)	
• Underweight (<18.5)	4 (1.72)
• Normal (18.5-24.9)	86 (36.91)
• Overweight (25–29.9)	73 (31.33)
• Obese (>30.0)	70 (30.04)
Smoking status, physican-reported, *n* (%)	
• Current smoker	8 (3.29)
• Ex-smoker	43 (17.70)
• Never smoked	192 (79.01)
Physician-reported pack years, *n* (%)	
• High (≥10)	29 (11.93)
• Low (<10)	22 (9.05)
• Never smoked	192 (79.01)
Lung function (FEV_1_)	
• Mean (SD)	73.65 (17.80)
• Range	25.0-138.0
• Median	70.0
• IQR	63.5, 82.0
Frequency of asthma exacerbations in last 12 months	
• Mean (SD)	1.08 (1.90)
• Range	0.0-14.0
• Median	0.0
• Range	0.0, 2.0
Frequency of asthma exacerbations in last 12 months, *n* (%)	
• 0	134 (55.14)
• 1	44 (18.11)
• ≥2	65 (26.75)
Deyo Charlson Comorbidity Index^a^ [58]	
• Mean (SD)	0.13 (0.53)
• Range	0-6
• Median	0
• IQR	0, 0
Most common concomitant conditions, *n* (%)	
• Allergic rhinitis	115 (47.33)
• Gastroesophageal reflux disease	42 (18.26)
• Anxiety	36 (15.65)
• Obesity	27 (11.74)
• Cardiovascular disease^c^	15 (6.17)
• None	45 (18.52)
Physician managing patient’s asthma, *n* (%)	
• Primary care- only	69 (28.40)
• Specialist-led	174 (71.60)
Morisky Medication Adherence Scale, *n* (%)	
• Low	97 (39.92)
• Medium	89 (36.63)
• High	57 (23.46)
Asthma Control Test	
• Mean (SD)	19.91 (3.94)
• Range	6-25
• Median	20
• IQR	18, 23
Asthma Control Test, *n* (%)	
• 5-19 (Not well controlled)	99 (40.74)
• ≥20 (Well controlled)	144 (59.26)
Jenkins Sleep Evaluation Questionnaire^b^	
• Mean (SD)	4.02 (4.53)
• Range	0-20
• Median	3
• IQR	0, 6
Jenkins Sleep Evaluation Questionnaire^b^, *n* (%)	
• 0	76 (31.28)
• 1-5	97 (39.92)
• 6-10	44 (18.11)
• 11-15	21 (8.64)
• 16-20	5 (2.06)
EuroQol-5D-3L	
• Mean (SD)	0.91 (0.14)
• Range	0.27-1
• Median	1
• IQR	0.84, 1

^a^Conditions as described by Deyo–Charlston index [57] are mapped from as many as 10 reported ICD-9-CM secondary diagnosis codes. A single summary cumulative value is represented. A score of 0 represents no comorbidities. Out of the 17 conditions, this research could accommodate 12 including: myocardial infarction, congestive heart failure, peripheral vascular disease, dementia, diabetes mellitus, cerebrovascular disease, COPD, connective tissue disease, mild liver disease, ulcer diagnosis, moderate or severe renal disease, any malignancy including lymphoma and leukemia, and acquired immunodeficiency syndrome. Missing data for a specific variable on the physician-reported form or the patient-reported from resulted in exclusion of the subject from the individual analysis for that variable but inclusion on all analyses for which data were not missing

^b^Higher score on the JSEQ indicates greater levels of sleep disturbance

^c^Cardiovascular disease excludes hypertension and hyperlipidaemia. FEV_1_, forced expiratory volume in 1 s; IQR, interquartile range; SD, standard deviation

Direct paths were hypothesized between 1) patient-reported inhaler satisfaction with drug delivery attributes, device functionality attributes, and device feedback attributes and adherence, 2) adherence and clinical outcomes, 3) comorbid AR and adherence, 4) comorbid AR and clinical outcomes, 5) smoking history and clinical outcomes, 6) smoking history and satisfaction with inhaler device drug delivery attributes, and 7) satisfaction with inhaler device drug delivery attributes and clinical outcomes. Our hypotheses about the relationships among the latent variables were based on expert consensus and interpretation of results from previous administrations of the DSP. Of the 3 domains associated with satisfaction, we hypothesized that only inhaler device drug delivery attributes would have a direct path to clinical outcomes. This was based on our assumption that satisfactory drug delivery would have a direct effect on clinical outcomes because drug delivery is most likely to be associated with symptom improvements.

## Results

**Fig. 1:**
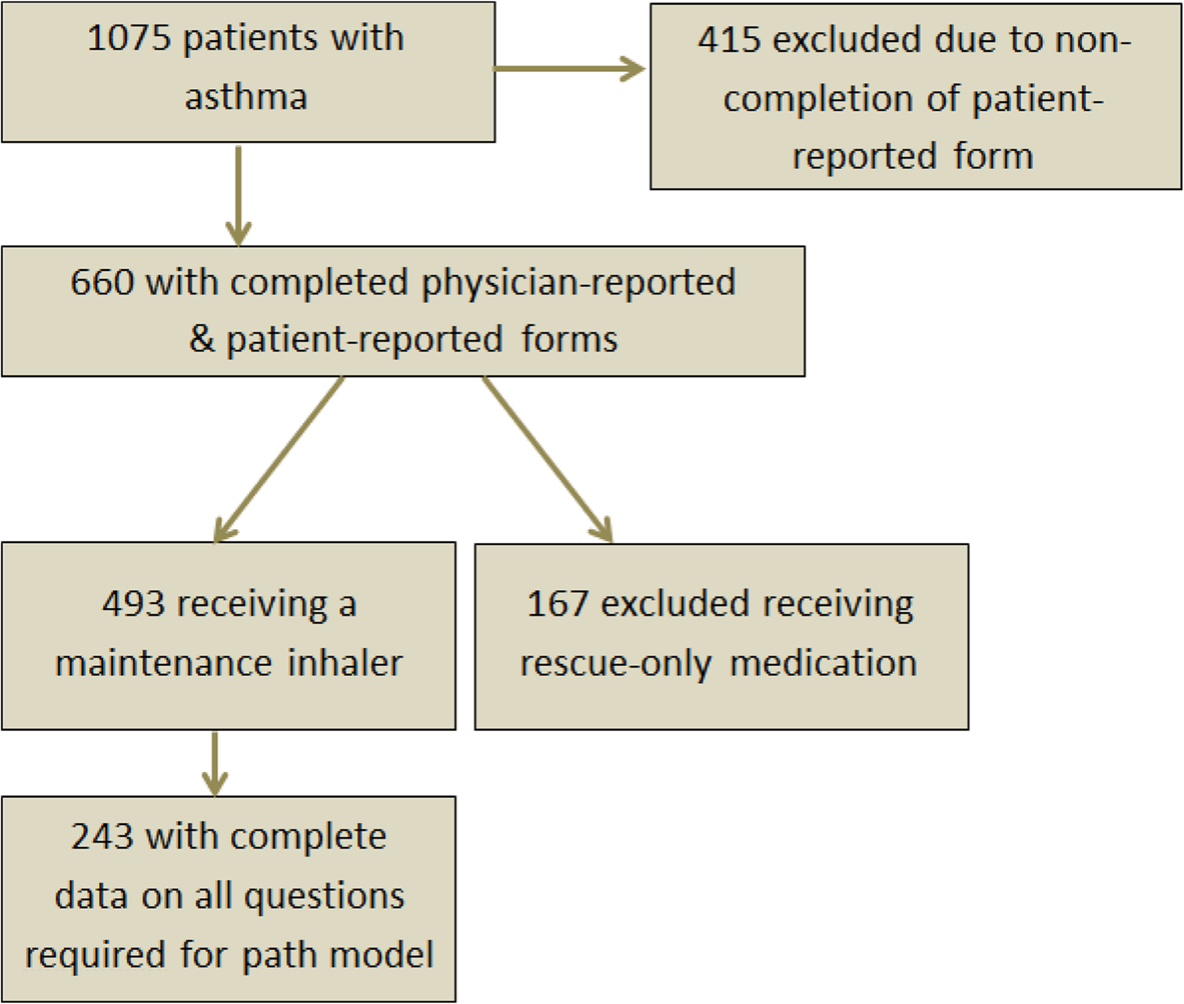
Patient study cohort

A total of 1075 asthma patients were recruited. Physician-completed forms and patient-completed forms were obtained for 660 of the 1075 patients, with 493 of these prescribed maintenance inhaler therapy. Of these, 243 patients were eligible for this analysis with complete data on all questions required for the path model (Fig. 1). A total of 209 physicians completed at least 1 physician-reported form, including 90 primary care physicians, 89 pulmonologists, and 30 allergists. Demographic and clinical characteristics of patients included in the model are summarized in Table 1, with 20.99 % (n = 51) reported to be current (3.29 %; n = 8) or prior smokers (17.70 %; n = 43). Almost half (47.33 %; n = 115) had comorbid AR.

### Patient outcomes

Overall, 41 % of patients had poorly controlled asthma based on the ACT with 45 % reported to have had 1 or more exacerbation and a mean of 1.1 exacerbations in the preceding 12 months. The mean JSEQ score was 4.0, equivalent to patient-reported sleep disruptions at least 1 night per week on average; 29 % of patients had JSEQ scores of 6 or higher. Overall patient-reported health status was high based on a mean EQ-5D-3L score of 0.9 (Table 2).

### Device satisfaction

Patients reported the highest level of satisfaction for attributes associated with inhaler device functionality, with scores ranging from 3.87 to 4.05 for 4 of 5 dimensions. Receiving the same amount of medicine to the lungs with each use received the highest ranking among drug delivery attributes and information about the number of doses remaining was the most highly-rated attribute for device feedback (Table 3). High satisfaction scores were also evident for the device functionality attributes of simple, easy-to-use instructions for the device, no need to put medication in the device prior to use, device durability, and ease of device portability.

**Table 3:** Patient-reported satisfaction with categories of inhaler device attributes

	Rating^a^
**Drug Delivery Satisfaction**	
I get the same amount of medicine delivered to my lungs each time	
• Mean (SD)	3.79 (0.88)
• Range	1-5
• Median	4
• IQR	3, 4
I do not need to breathe in hard to inhale my medicine	
• Mean (SD)	3.58 (1.02)
• Range	1-5
• Median	4
• IQR	3, 4
Low/no irritation in mouth and throat	
• Mean (SD)	3.45 (1.07)
• Range	1-5
• Median	3
• IQR	3, 4
I do not need to breathe in at the same time as I press my inhaler	
• Mean (SD)	3.42 (1.12)
• Range	1-5
• Median	3
• IQR	3, 4
**Device Functionality**	
The instructions are simple and easy to use	
• Mean (SD)	4.05 (0.83)
• Range	1-5
• Median	4
• IQR	4, 5
It is built to last and will not break easily	
• Mean (SD)	3.94 (0.83)
• Range	1-5
• Median	4
• IQR	3, 5
No need for me to put the medicine in the inhaler before I use it	
• Mean (SD)	3.97 (0.95)
• Range	1-5
• Median	4
• IQR	3, 5
Easy to hold and carry around with me	
• Mean (SD)	3.87 (0.92)
• Range	1-5
• Median	4
• IQR	3, 5
Can reuse the inhaler for more than one month	
• Mean (SD)	2.89 (1.36)
• Range	1-5
• Median	3
• IQR	2, 4
**Device Feedback Satisfaction**	
It tells me how many doses of medicine I have left	
• Mean (SD)	3.72 (1.14)
• Range	1-5
• Median	4
• IQR	3, 5
The inhaler locks when empty so it cannot be used anymore	
• Mean (SD)	3.08 (1.32)
• Range	1-5
• Median	3
• IQR	2, 4
It tells me when my dose of medicine has been inhaled correctly	
• Mean (SD)	3.06 (1.27)
• Range	1-5
• Median	3
• IQR	2, 4

^a^1 = not at all satisfied; 5 = very satisfied. SD, standard deviation. IQR, interquartile range; SD, standard deviation

Lower levels of satisfaction were reported for the attributes of being usable for more than 1 month, providing feedback that the medication dose was correctly inhaled, and device locking to prevent use of an empty inhaler.

Scores on the MMAS-8 revealed that 24 % (*n* = 57) of patients demonstrated high adherence to their medication regimen, 37 % (n = 89) medium or moderate adherence, and 40 % (n = 97) had low adherence.

### Path modeling for patient outcomes

Cronbach’s alpha [Cronbach 1951] provides a measure of consistency for a group of variables. Cronbach’s alphas of 0.81 for inhaler device drug delivery satisfaction, 0.83 for inhaler device functionality satisfaction, 0.75 for inhaler device feedback satisfaction, 1.00 for comorbid AR, 1.00 for adherence, 0.91 for smoking history, and 0.713 for outcomes indicated unidimensionality of the manifest variables for each latent variable. Unidimensionality demonstrates that variables are moving in the same direction, which implies they are manifestations of the same underlying latent variable. Cross-loadings or correlations revealed that all manifest variables were associated with appropriate latent variables in the hypothesized model.

**Fig. 2:**
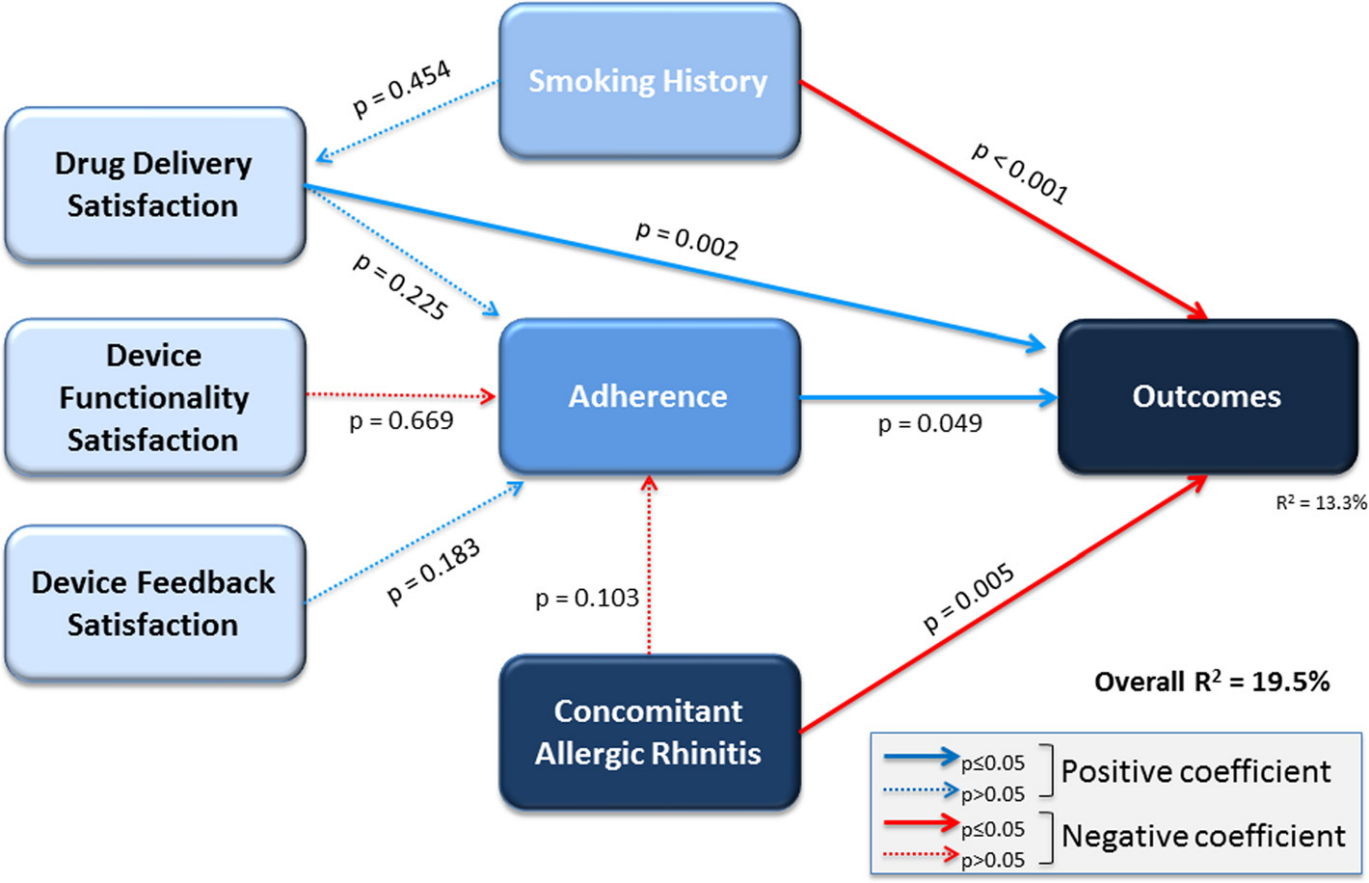
Partial least squares path modeling analysis examining the relationship between inhaler device satisfaction, medication adherence, smoking history, allergic rhinitis, and patient outcomes

More favorable clinical outcomes were significantly associated with greater patient satisfaction with drug delivery (*P* = 0.002), higher medication adherence (*P* = 0.049), a negative history for tobacco use (*P* < 0.001), and no comorbid AR (*P* = 0.005). The R^2^ for outcomes (the proportion of variability in outcomes explained by the model) was 13.3 % and the pseudo goodness of fit (a measure of the overall prediction performance of the path model) was 19.5 % (Fig. 2).

## Discussion

Our findings confirm multiple factors are predictive of patients’ degree of asthma control, including satisfaction with attributes of inhaler devices, adherence, concomitant health conditions, and tobacco use. Notably, drug delivery attributes of the inhaler device and higher adherence to treatment were significantly associated with more favorable patient outcomes, including better asthma control, improved quality of sleep, better overall health status, and lower frequency of asthma exacerbations. While satisfaction with device functionality and device feedback were not significantly associated with clinical outcomes, the ability to properly use the device and understand device feedback are related to optimal delivery of medication and likely play a role in device satisfaction and improved asthma management. In contrast, concomitant AR and smoking history were significantly associated with poor asthma control.

While the 3 inhaler device satisfaction domains of delivery, functionality, and feedback were not significantly associated with treatment adherence, the relationship between satisfaction and adherence was in the expected direction of greater satisfaction resulting in better adherence. Additional research is needed to further refine our understanding of the relationship between different attributes of inhaler devices and adherence to treatment. However, the trend for higher rates of adherence among patients who were more satisfied with their inhaler device in our research is consistent with results from research examining the association between patient satisfaction with treatment regimens and adherence across diverse chronic health conditions [6, 29–32]. A systematic review revealed significant associations between higher treatment satisfaction and better treatment adherence and persistence for patients with glaucoma, diabetes, osteoporosis, or schizophrenia [29]. Two cross-sectional surveys of adults receiving medical therapy for chronic diseases revealed that dissatisfaction with their medication schedule, side effects, and the burden of receiving treatment were significant predictors of intentional nonadherence [30]. Among older patients with asthma, adjusted multivariate models revealed that treatment dissatisfaction (characterized by weak perceptions of the benefits and need for treatment, and treatment safety concerns) was an independent predictor of nonadherence [6]. In turn, medication adherence is predictive of clinical outcomes [45], with nonadherence to asthma medications associated with poor control, exacerbations, hospitalizations, and declines in lung function [46].

Attributes that were associated with device satisfaction in this study included patient perceptions of consistency in the amount of drug delivery to the lungs, ease of use, and feedback about the number of remaining doses. As reported in our results, the correlations revealed that all manifest variables were associated with the appropriate latent variables in the hypothesized model. This offers confirmation that the appropriate domains had been selected for the 12 device satisfaction attributes. While beyond the scope of our research, future investigations may expand our descriptive analysis of inhaler device attributes within the 3 domains to include statistical comparisons of differences between device attributes and their impact on patient outcomes.

Our analysis suggests that a direct relationship may exist between inhaler satisfaction and clinical outcomes. Therefore, clinicians may need to address issues related to patient satisfaction with inhaler devices in order to achieve optimal clinical outcomes. Specifically, clinicians’ choice of inhaler device and provision of patient education on optimal techniques for device utilization may increase patient satisfaction as well as adherence with device therapy [47, 48]. Alternative models might also predict relationships between behaviors, treatment, therapeutic adherence, and clinical outcomes in patients affected by asthma. This will be a fruitful area for future clinical and psychometric research to further clarify independent and correlated predictors of clinical outcomes, which has the potential to provide clinicians with information to improve the management of their patients with asthma.

Our findings suggest tobacco use and concomitant AR are associated with poorer clinical outcomes in patients with asthma. While the percentage of current smokers was low in our study at 3.29 %, it is possible that physicians underestimated current smoking rates in their patients due to patient inaccurately identifying themselves as never or past smokers. A cross-sectional study of patients with respiratory disorders revealed low concordance between self-reported tobacco use and objective measures of smoking. Among patients with asthma, 29 % of those who self-identified as nonsmokers or past smokers had objective measures indicative of current tobacco use [49]. These results suggest that patients frequently provide inaccurate information about their current smoking status to their clinicians. This could be a possible explanation for our finding that a past history of tobacco use was associated with less favorable clinical status because patients classified as past tobacco users had not actually discontinued smoking.

Furthermore, tobacco use and AR are both modifiable factors and have the potential to improve asthma control in patients with appropriately targeted therapy. For example, previous research has shown that tobacco use is associated with decreased efficacy of ICS-containing maintenance regimens [21, 23, 26]. Therefore, the choice of medical therapy may need to be tailored to patients who are current or past smokers. In particular, current smokers with asthma may require treatment with higher doses of traditional ICS, extra-fine ICS formulations to reduce small airway inflammation [50], leukotriene receptor antagonists, or combination therapy with extra-fine ICS and long-acting beta-agonists or leukotriene receptor antagonists [21]. The co-occurrence of AR in patients with asthma has been shown by other researchers to negatively affect patient outcomes [15, 16], with 65.7 % of patients with moderate-to-severe persistent AR experiencing inadequate asthma control due to increased inflammation of the lower airways [16]. Interventions to promote smoking cessation, tailored therapies for current or past smokers with asthma, and medical therapies to improve the management of AR may improve outcomes for patients with asthma [16, 51].

In addition to proper selection of medical therapies for patients with AR and those who are current or past users of tobacco products, our findings suggest that increased patient satisfaction with inhaler devices provides clinicians with a modifiable treatment option to improve clinical outcomes and quality of life for their patients with asthma. Proper use of inhaler devices and patient adherence with medical therapies for asthma are suboptimal, with a wide variety of factors contributing to improper use of devices, satisfaction with inhalers, and medication adherence [10, 52, 53]. More efficient use of inhaler devices, better device technology to ensure drug delivery, and device design that promotes adherence have the potential to improve control of asthma [54].

Several limitations should be considered in the evaluation of our findings. We assessed a number of patient and clinical variables but this was not an exhaustive list of all factors that might influence clinical outcomes in patients with asthma. Additional factors associated with suboptimal asthma control include older patient age, lower socioeconomic status, the failure to recognize symptoms associated with exacerbations, lack of asthma self-management education [55], poor recognition of asthma triggers [56], and poor disease knowledge [57]. Future research may include these variables as well as treatment adherence and satisfaction to identify patients at risk for poor disease control. The cross-sectional design of this study prevents any conclusions about causal relationships between these variables and patients’ clinical outcomes, which could potentially be evaluated in a longitudinal study.

The proportion of variability in outcomes explained by the model was 13.3 % and the pseudo goodness of fit was 19.5 %. Although these values are not particularly high, it is not unusual to obtain lower values for R^2^ or goodness of fit when predicting human behavior. The demonstration of statistically significant relationships is of interest and potentially of clinical value.

Furthermore, the DSP was not based on a true random sample of physicians or patients. While minimal inclusion criteria governed the selection of the participating physicians, participation might have been influenced by willingness to complete the survey and practical considerations of location. We were not able to evaluate differences in our results attributable to physician specialty. We recognize that the level of knowledge and management strategies for asthma might differ between primary care physicians and pulmonologists and such differences might affect treatment satisfaction and clinical outcomes. This presents an opportunity for exploration in future research. Fully completed physician-reported forms and patient-reported forms were obtained for 243 of the 493 eligible patients, equivalent to a 49 % response rate. It is unclear whether this may have introduced a bias to our results.

While our research design included methods to ensure that physicians and staff were unaware of patient responses on the patient-reported forms, it was not possible to confirm that no information exchange occurred between physicians and their patients. This has the potential to undermine the accuracy of estimated treatment adherence as well as patient responses to the standardized PRO questionnaires and the assessment of inhaler device satisfaction. Recall bias might also have affected the responses of both patients and physicians to the questionnaires, which is a common limitation of surveys. However, the data for these analyses were collected at the time of each patient’s appointment and this is expected to reduce the likelihood of recall bias.

Despite these limitations, our findings provide valuable real-world physician- and patient-matched results suggesting that proper selection of inhalation devices and patient education combined with improved medication adherence, optimal treatment of AR and smoking cessation and/or tailoring medical therapy for current smokers, may lead to more favorable clinical outcomes. Addressing one or all of these areas has potential to decrease patients’ risk of exacerbations and improve asthma control and overall quality of life.

## Conclusions

Significant predictors of better asthma control, improved quality of sleep, better overall health status, and lower frequency of asthma exacerbations were associated with greater treatment adherence and patient satisfaction with attributes of their inhaler device, including perceptions of consistency in the amount of drug delivery to the lungs, ease of use, and feedback about the number of remaining doses. AR and history of tobacco use were also significantly and negatively associated with disease control. These findings provide clinicians with strategies that can be tailored to the individual patient to improve clinical outcomes. Specifically, choice of inhaler device and provision of patient education to ensure optimal use of the device as well as treatment of concomitant AR and consideration of the patient’s smoking history are modifiable factors that may optimize asthma control and improve the quality of life for patients with asthma.
